# Targeting Nanotherapeutics for Highly Efficient Diagnosis and Treatment of Systemic Lupus Erythematosus through Regulation of Immune Response

**DOI:** 10.1002/smsc.202400521

**Published:** 2025-01-23

**Authors:** Ting Liu, Zhiming Lin, Xi Zhang, Yu Yang, Guanning Huang, Yanzi Yu, Bin Xie, Lizhen He, Tianfeng Chen

**Affiliations:** ^1^ The Department of Chemistry Department of Neurology and Stroke Center of The First Affliated Hospital State Key Laboratory of Bioactive Molecules and Druggablily Assessment MOE Key Laboratory of Viral Pathogenesis and lnfection Prevention and Control Jinan University Guangzhou 510632 China; ^2^ Division of Rheumatology Third Affiliated Hospital of Sun Yat‐Sen University Guangzhou 510630 China

**Keywords:** anti‐dsDNA antibodies, regulations of immune response, specific targeting, systemic lupus erythematosus

## Abstract

Systemic lupus erythematosus (SLE) is characterized by the production of pathogenic autoantibodies, particularly antidouble‐stranded (ds) DNA antibodies, which contribute to multiorgan damage (lupus nephritis, LN). Hence, there is an urgent need to recognize and eliminate SLE‐specific anti‐ds DNA antibodies to enhance the SLE treatment. Herein, mesoporous silica (MSNs) loaded with SeC and surface‐modified ctDNA are constructed to effectively specific bind and eliminate pathogenic anti‐dsDNA antibodies for treatment SLE in 125 plasm and enabling swift LN diagnosis in 36 kidney tissue from SLE patients. As expected, the clearance ratio of anti‐dsDNA antibodies by nanotherapeutics is significantly greater compared to other products commonly used in clinical therapies and exhibits biocompatibility and safety in patients. Moreover, MSNs‐DNA can also help visualize the distribution of anti‐dsDNA antibodies in the lesions of the kidney. Importantly, the combination strategy (MSNs‐DNA@SeC) can effectively remove antibodies and reduce UP production by the regulation of B cells and T cells in female MRL/lpr SLE model mice to alleviate related symptoms. Collectively, the resultant data not only presents a straightforward method for the systematic design of nanomedicine targeting SLE to enhance the effects on diagnosis and treatment, but also elucidates the potential mechanisms involving anti‐dsDNA antibodies in the pathogenesis and progression of SLE.

## Graphical abstract

Systemic lupus erythematosus (SLE) is characterized by the production of pathogenic autoantibodies, particularly antidouble‐stranded (ds) DNA antibodies, which contribute to multiorgan damage (lupus nephritis, LN). Herein, mesoporous silica loaded with SeC and surface‐modified ctDNA are constructed to target binding anti‐dsDNA antibodies, effectively eliminating pathogenic antibodies, alleviating symptoms, enabling swift LN diagnosis, and introducing innovative strategies for managing SLE.

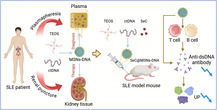

## 1 Introduction

As an autoimmune connective tissue disease, the etiology of systemic lupus erythematosus (SLE) remains unknown.^[^ ^1^, ^2^ ^]^ It encompasses a heterogeneous spectrum of disorders distinguished by the breakdown of immune tolerance toward multiple autoantigens and the aberrant modulation of both innate and adaptive immune responses.^[^ ^3^ ^]^ Notably, patients afflicted with SLE prominently exhibit the generation of diverse pathogenic autoantibodies and immune complexes, inciting widespread damage across numerous anatomical systems and organs, particularly in individuals afflicted with severe manifestations of the ailment.^[^ ^4^, ^5^ ^]^ Numerous studies have underscored the significance of antidouble‐stranded (ds) DNA antibodies as significant contributors to immune dysregulation and initiators of organ damage in SLE.^[^ ^6^ ^]^ Moreover, these antibodies hold widespread utility as serological markers for diagnosing SLE and evaluating disease activity in clinical practice.^[^ ^7^ ^]^ Thus, it is evident that a crucial role can be contributed by anti‐dsDNA antibodies in the onset and progression of SLE.

Specifically, anti‐dsDNA antibodies frequently accumulate in the kidneys of SLE patients, leading to kidney damage and the development of lupus nephritis (LN).^[^ ^8^, ^9^ ^]^ LN manifests in around half of individuals diagnosed with SLE and represents a noteworthy factor in mortality rates.^[^ ^10^, ^11^, ^12^ ^]^ However, traditional techniques (e.g., immunofluorescence staining, hematoxylin‐eosin (HE)) to distinguish LN from other kidney diseases are still challenging.^[^ ^13^, ^14^ ^]^ Hence, there is a need to develop a novel nanosystem that can identify and label anti‐dsDNA antibodies accurately and directly, particularly in the tissues of kidney, to enhance LN diagnosis.

The clinical manifestations of SLE vary widely, leading to the widespread use of glucocorticoids and immunosuppressants in its treatment. However, these therapies fail to swiftly reduce autoantibodies accumulation, including anti‐dsDNA antibodies.^[^ ^15^, ^16^ ^]^ Currently, plasma adsorption columns are commonly employed to eliminate therapeutic antibodies from peripheral blood and alleviate patient symptoms.^[^ ^17^, ^18^ ^]^ Nevertheless, their effectiveness is limited, and they do not directly counteract the production of pathogenic antibodies. Therefore, there is an urgent need for more reliable treatments that specifically target the removal of anti‐dsDNA antibodies and alleviate symptoms to improve SLE management.

**Scheme 1:**
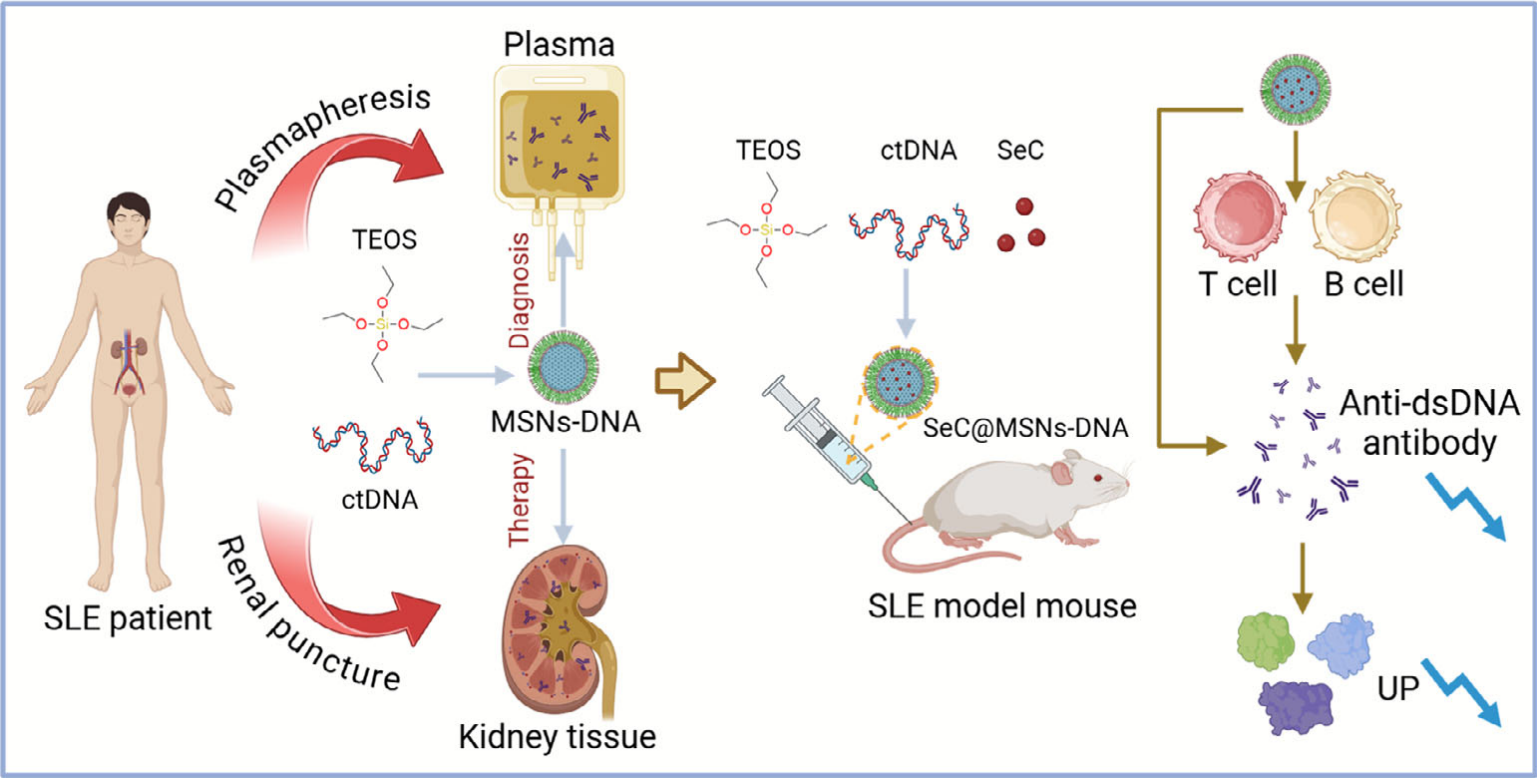
Schematic illustration of SeC@MSNs‐DNA nanoparticles for highly efficient diagnosis and treatment of SLE in vivo by regulation of B cells and T cells.

The emergence of nanomaterials has revolutionized disease diagnosis and treatment, bringing forth breakthroughs and fostering hope. Recently, the utilization of nanomaterials in various aspects of disease diagnosis and treatment has gained increasing attention.^[^ ^19^, ^20^, ^21^ ^]^ In recent years, the role of nanomaterials in immune modulation has captured the interest of many researchers, encompassing study areas like nanovaccines and the implications of nanomaterials in autoimmune diseases.^[^ ^22^, ^23^, ^24^ ^]^ Among the range of nanomaterials available, selenium (Se) nanomaterials are distinguished for their unique properties. Se, an essential trace element for human health, fulfills a critical role in regulating diverse biological processes, including thyroid hormone metabolism, cardiovascular health, and immune function, when integrated into selenoproteins such as thioredoxin reductase (TrxR) and glutathione peroxidase (GPx).^[^ ^25^, ^26^, ^27^ ^]^ It also indicates varying demands of Se for reactive oxygen species (ROS) and critical intracellular antioxidant pathways, such as those involving components of the thioredoxin (Trx) and glutathione (GSH) systems, crucial for the proliferation and viability of immune cells.^[^ ^28^, ^29^ ^]^ Furthermore, it was found that Se supplementation can boost the regulation and differentiation of T cells in human.^[^ ^30^, ^31^ ^]^ Chen et al. revealed that Se can heighten the activity of natural killer (NK) cells by modulating surface markers, related pathways, and intracellular redox balance.^[^ ^32^, ^33^ ^]^ Se can also adjust the redox equilibrium within the tumor microenvironment through TrxR1 to influence the response of immunity.^[^ ^34^ ^]^ In this study, mesoporous silica (MSNs) was utilized as a carrier, loaded with SeC, and surface‐modified ctDNA to target binding anti‐dsDNA antibodies, effectively eliminating pathogenic antibodies, alleviating symptoms, enabling swift LN diagnosis, and introducing innovative strategies for managing SLE (**Scheme** **1**).

## 2 Results and Discussions

### 2.1 Rational Design of MSNs‐DNA Targeting for Anti‐dsDNA Antibodies

**Figure 1:**
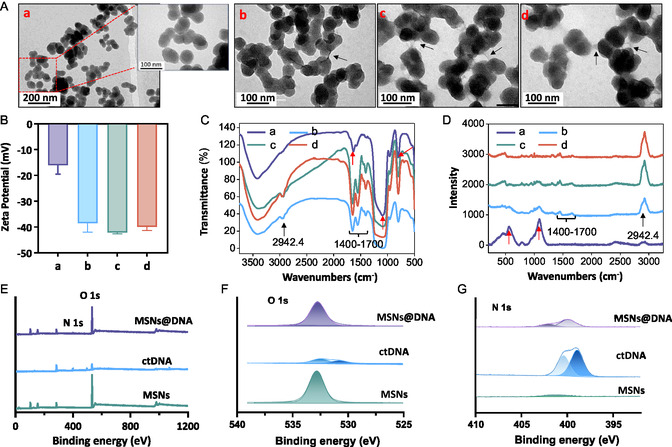
Characterization of MSNs‐DNA before and after adsorbed anti‐dsDNA antibodies from SLE patients’ plasma. Changes in A) TEM image, B) zeta potential, C) FT‐IR spectra, and D) Raman spectra of MSNs‐DNA before and after adsorbing anti‐dsDNA antibodies from SLE patients’ plasma (a: before adsorbing the anti‐dsDNA antibodies; b,c, and d: after adsorbing the anti‐dsDNA antibodies from different SLE patients’ plasma). E) XPS spectra, F) O 1*s* spectra, and G) N 1*s* spectra of MSNs, ctDNA, and MSNs‐DNA.

Herein, we designed that MSNs were decorated by ctDNA (MSNs‐DNA) for specifically combination anti‐dsDNA antibodies to eliminate antibodies directly in SLE patients. First, MSNs‐DNA nanoparticles were successfully created and characterized by microscopic and spectroscopic analysis (**Figure** **1**). From the transmission electron microscopy (TEM) image, we found that MSNs‐DNA were spherical nanoparticles with hydrated size 161.3 nm and zeta potential −16 mV (Figure 1A,B and S1, Supporting Information). Fourier transform infrared spectroscopy (FT‐IR) and Raman spectroscopy of MSNs‐DNA showed the peak of Si—O—Si at 1098 and 1090 cm^−1^, the peak of 800 and 553 belonging to MSNs, and the peak of —NH_2_ at 1637 cm^−1^, which belonged to ctDNA (Figure 1C,D). Importantly, from the results of XPS, we found (Figure 1E–G) that there was no N signal in MSNs, and the signal of O was lower than in MSNs and MSN@DNA. Collectively, these results indicated that MSNs‐DNA were created as designed.

Furthermore, we also examined the changes of MSNs‐DNA after adsorbing anti‐dsDNA antibodies from SLE patients’ plasma. As shown in Figure 1A, the surface layer of nanoparticles absorbed some protein substances from SLE patients’ plasma, and some cross‐linked substances appeared between the nanoparticles. In addition, the zeta potential of MSNs‐DNA after treatment decreased from −16 mV to about −40 mV (Figure 1B). Moreover, FT‐IR spectra and Raman spectroscopy of MSNs‐DNA after adsorbing anti‐dsDNA antibodies from SLE patients’ plasma showed the peak at 1400–1700 and 2942.4 cm^−1^, which belonged to —CONH— and —OH in antibodies, respectively (Figure 1C,D). These findings indicated the successful synthesis of MSNs‐DNA and the effective elimination of antibodies by MSNs‐DNA.

### 2.2 The Adsorption Process of SLE Patient's Plasma on MSNs‐DNA

**Figure 2:**
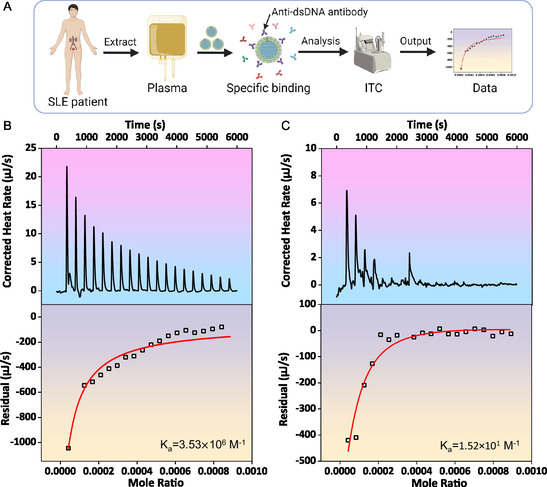
ITC data for the adsorption process of SLE patient's plasma on MSNs‐DNA. A) The adsorption process of SLE patient's plasma on MSNs‐DNA by ITC. B) High clearance rate and C) low clearance rate of anti‐dsDNA antibodies in the plasma of SLE patients by MSNs‐DNA. The upper graphs display the raw titration data, while the lower graphs present the integrated energy of each peak alongside the corresponding fitting curve.

We further investigated the adsorption process of SLE patient's plasma on MSNs‐DNA by isothermal titration calorimetry (ITC) assay. Despite both MSNs‐DNA and antibodies being negatively charged under identical conditions, the binding of antibodies is enhanced by electrostatic attraction via their positively charged regions (**Figure** **2**A). In order to prove that MSNs‐DNA can specifically bind to anti‐dsDNA antibodies, we selected plasma from high titers and low titers of anti‐dsDNA antibody patients to interact with MSNs‐DNA in the ITC assay, respectively. As in Figure 2B,C, the binding affinity of SLE patient's plasma with high anti‐dsDNA titer to MSNs‐DNA was stronger than SLE patient's plasma with low anti‐dsDNA titer, which was verified by Ka = 3.53 × 10^6^ and Ka = 1.52 × 10^1^ m ^−1^ in the corresponding titration. The values obtained from the ITC analysis suggest a robust interaction between anti‐dsDNA antibodies and MSNs‐DNA, thereby reinforcing the notion of a potent electrostatic interaction. These results indicate that MSNs‐DNA could specifically recognize and bind anti‐dsDNA antibodies in plasm from patients.

### 2.3 MSNs‐DNA Achieved the Clearance Ability against Antibodies

**Figure 3:**
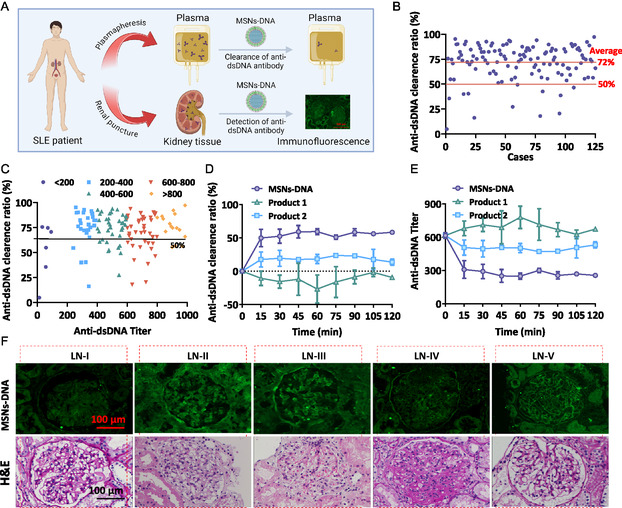
The clearance ability against antibodies by MSNs‐DNA and immunofluorescence on kidney biopsy from lupus nephritis patients stained by MSNs‐DNA. A) Graphic scheme showing the clearance of antibodies and LN diagnosis process using MSNs‐DNA. B,C) The clearance ability against antibodies of MSNs‐DNA in 125 cases with MSNs‐DNA and plasma coincubated for 2 h. D) The clearance ability against antibodies by MSNs‐DNA, product 1, and product 2 and E) the titer of plasma after MSNs‐DNA, product 1, and product 2 coincubated with plasma for 2 h. F) H&E and immunofluorescence of kidney tissue in LN I patient, LN II patient, LN III patient, LN IV patient, and LN V patient. Each value represents means ± SD (*n* = 3).

Then, we examined the clearance ratio of anti‐dsDNA antibodies by MSNs‐DNA to assess the clearance ability against antibodies in **Figure** **3**A. The ELISA kit was employed to assess the anti‐dsDNA antibody titers in the plasma of 125 SLE patients before and after absorption by MSNs‐DNA. As shown in Figure 3B, the average clearance ratio was 72%, and the clearance rate in most cases is greater than 50%. Moreover, the anti‐dsDNA antibodies titer in most cases is greater than 200, and the clearance rate was also above 50% (Figure 3C). In order to further verify the anti‐dsDNA antibodies clearance rate by MSNs‐DNA, we compared the clearance ratio of MSNs‐DNA with plasma adsorption columns on the market product 1 and product 2. It was found that the clearance ratio of MSNs‐DNA was significantly higher than that of product 1 and product 2 (Figure 3D). At the same time, the results of plasma titers after adsorption were also consistent with the clearance ratio (Figure 3E). Collectively, our data demonstrated that the as‐synthesized MSNs‐DNA could effectively remove anti‐dsDNA antibodies in plasma from SLE patients.

### 2.4 Immunofluorescence on Kidney Tissues by MSNs‐DNA in LN Patients

To investigate the biodistribution of anti‐dsDNA antibodies on renal tissue, we used fluorescently labeled nanoparticles to incubate the kidney tissue section for observing antibodies distribution by fluorescence microscope (Figure 3A). As shown in Figure 3F, the image of H&E demonstrated nearly normal appearance of glomerulus from LN I, while we observed mesangial immune deposits after treatment by MSNs‐DNA. H&E section from LN II patient showed mesangial matrix expansion or purely mesangial hypercellularity, but mesangial immune deposits when incubated with MSNs‐DNA (Figure 3F). After tissue from an LN III patient was stained by MSNs‐DNA, segmental or global endocapillary or extracapillary glomes were shown positively, active or inactive focal and with or without mesangial alterations (Figure 3F). The H&E results in LN IV patients showed glomerulonephritis in more than half of glomeruli, after treatment with MSNs‐DNA could observe subendothelial immune deposition in renal tissue (Figure 3F). The results of H&E and immunofluorescence both showed their morphological sequelae or global or segmental subepithelial immune deposits in patients with LN V (Figure 3F). These results indicated that the lesions observed in immunofluorescence are consistent with the H&E results. Therefore, we believed that fluorescently labeled MSNs‐DNA can be used to detect the biodistribution against anti‐dsDNA antibodies in the kidneys of SLE patients.

### 2.5 Evaluation of the Biocompatibility and Safety of MSNs‐DNA

**Figure 4:**
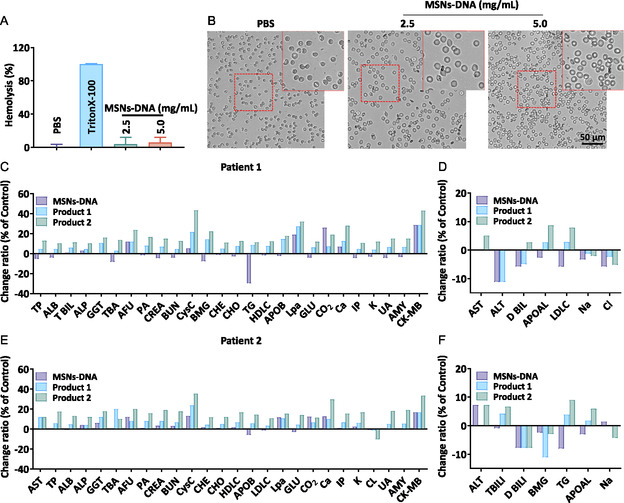
Evaluation the biocompatibility and safety of MSNs‐DNA. A) The hemolysis rate of erythrocyte after treatment with MSNs‐DNA and B) the morphology changes of erythrocyte. Blood biochemistry data with C) significant change and D) slight change in plasma of SLE patient 1. Blood biochemistry data with E) significant change and F) slight change in plasma of SLE patient 2 before and after adsorption. Each value represents means ± SD (*n* = 3).

In the clinic, the purified plasma will be returned to the patients, thereby the safety and biocompatibility of MSNs‐DNA is a vital factor limiting future applications. Firstly, we carried out hemocompatibility assay of MSNs‐DNA to evaluate the biocompatibility and safety of MSNs‐DNA. As shown in **Figure** **4**A, the hemolysis of MSNs‐DNA was less than 5%. Compared with the control group, there was no change in the erythrocyte morphology after MSNs‐DNA treatment (Figure 4B). Then, we further investigated the changes in blood biochemical indexes after MSNs‐DNA treatment. From Figure 4C,D, we could find that 26 indexes significantly increased after product 2 treatment in patient 1, while MSNs‐DNA treatment only caused changes in a few indicators. In patient 2, there were also 26 indexes significantly elevated after product 2 treatment, while there were few indexes changed after MSNs‐DNA treatment. Next, we examined the effect of ALB, K, Na, and Cl with MSNs‐DNA, product 1, and product 2 treatment in patients 3 and 4, and we have noticed no significant changes (Figure S3, Supporting Information). Collectively, these data demonstrate that MSNs‐DNA exhibit good biocompatibility and safety.

### 2.6 A Safe and Effective Combination Strategy Demonstrates High Therapeutic Efficacy against SLE in MRL/lpr Mouse

**Figure 5:**
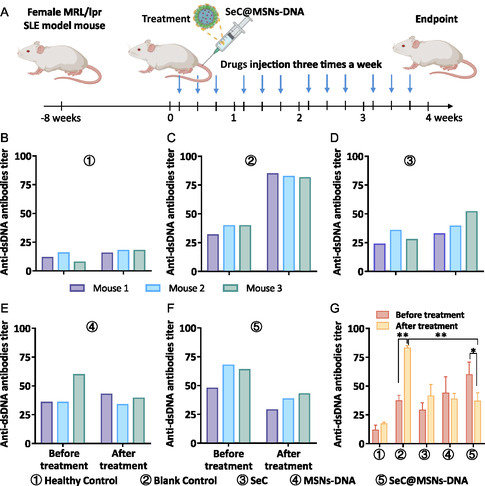
Anti‐dsDNA antibodies in MRL/lpr SLE model mouse of MSNs‐DNA. A) The scheme of the treatment process in MRL/lpr SLE model mouse. B–G) The anti‐dsDNA antibodies in MRL/lpr SLE model mouse before and after treatment. Each value represents means ± SD (*n* = 3). *P < 0.05, **P < 0.01, ***P < 0.001.

In order to prove that MSNs‐DNA can also specifically eliminate anti‐dsDNA antibodies and alleviate the symptoms of SLE in vivo, therefore, we next set out to combine SeC to MSNs‐DNA nanoplatform (MSNs‐DNA@SeC) against SLE in MRL/lpr mouse and evaluated the therapeutic efficacy of DNA‐SeNPs in female MRL/lpr SLE model mouse (**Figure** **5**A). As shown in Figure 5B,G, there was no change in the titer of anti‐dsDNA antibodies in 21 days. Compared to the Healthy Control “HC” group, the titer of anti‐dsDNA antibodies was significantly increased in SLE control (Figure 5C,G). At the same time, the titer of anti‐dsDNA antibodies was slightly increased and decreased after treatment with SeC and MSNs‐DNA, respectively (Figure 5D,E,G). We found MSNs‐DNA@SeC could effectively eliminate anti‐dsDNA antibodies in the plasm of female MRL/lpr SLE model mouse (Figure 5F,G). A prominent feature of SLE patients is elevated urinary protein (UP).^[^ ^35^, ^36^ ^]^ UP is an important indicator for evaluating kidney function; therefore, we further examined the urinary protein content in mouse urine. Mouse urine samples with protein ≥3 g L^−1^ was designated as severe proteinuria. Before treatment, the UP content increased to 3 g L^−1^, which indicated that kidney disease induced by SLE (Table S3, Supporting Information). After treatment, we found the UP decreased to 0.3 and 0 g L^−1^, that of SLE control was 3.0 g L^−1^ (Table S3, Supporting Information), suggesting that UP in the “HC” group remained at a relatively low level throughout the treatment process, while UP levels in the final treatment group decreased or remained unchanged. These results of UP and serum antibodies showed the same trend of improvement in lupus symptoms. Furthermore, there was no significant change in body weight between the control and treated groups in Figure S4, Supporting Information, suggesting the safety of the combination therapeutic strategy. Overall, these results demonstrated that a combination strategy could effectively remove antibodies and reduce UP production from the SLE mouse and alleviate related symptoms.

**Figure 6:**
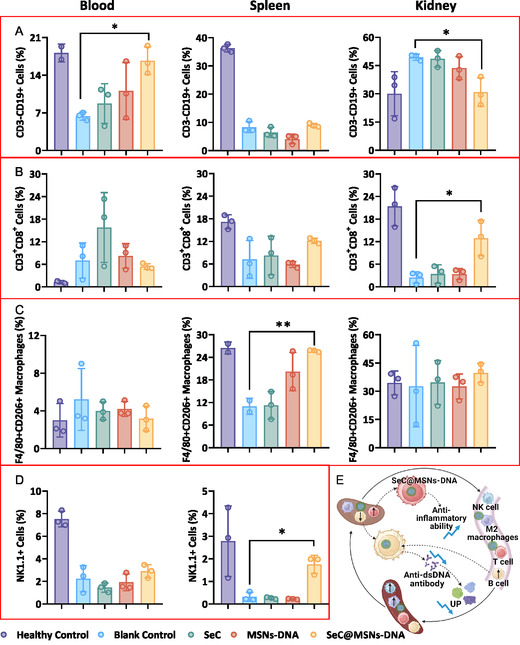
Regulation of immune response by SeC@MSNs‐DNA in MRL/lpr SLE model mouse. Representative flow cytometry plots showing the ratio of A) CD^3−^CD19^+^ B cells, B) CD8^+^ T cells, C) M2‐macrophage (CD206^+^F4/80^+^ in CD11b^+^CD45^+^ cells), and D) NK cells separate in spleen, blood, and kidney from different treated mice. E) Regulation of immune response by SeC@MSNs‐DNA in MRL/lpr SLE model mouse. Each value represents means ± SD (*n* = 3). *P < 0.05, **P < 0.01, ***P < 0.001.

Previous study found a significantly higher frequency of CD19^+^ B cells in SLE patients compared to the HC group and with a positive correlation with disease activity.^[^ ^37^ ^]^ Our results show a significant decrease in CD3^+^CD19^+^ B cells in both the blood and spleen of the SLE group compared to the HC group, while an increase was observed in the kidney of the SLE group. (**Figure** **6**A). Furthermore, we found that CD^3+^CD19^+^ B cells in the blood of SLE mice significantly increased after treatment with MSNs‐DNA@SeC, whereas there was no change in the spleen, but they decreased in the kidney, compared to the SLE group (Figure 6A). Therefore, treatment promoted the tendency of B cells to mature benignly in the blood. Additionally, it is verified that B cells may play a role in the occurrence and development of SLE, which provides a direction for cell‐targeted therapy of SLE diseases in the future.

We also analyzed the other immune cells in the spleen, blood, and kidney by flow cytometry. Among them, the CD8^+^ positive T cells in the blood of the SLE group were higher than those of the HC group, but both in the spleen and kidney of the SLE group were lower than those of the HC group (Figure 6B). Compared with the SLE group, after SeC@MSNs‐DNA treatment, T cells were almost unchanged in the blood, and T cells both in the spleen and kidney of SLE mice increased, especially in the kidney (Figure 6B). Compared to the HC group, M2 macrophages increased in the SLE group, while in the spleen significantly decreased, and were almost unchanged in the kidney (Figure 6C). On the other hand, compared with the SLE group, M2 macrophages decreased in the blood, while they significantly increased in the spleen, and were almost unchanged in the kidney with MSNs‐DNA@SeC treatment (Figure 6C). NK cells were lower in the SLE group than in the HC group both in the spleen and blood, while in the spleen was significantly increased after MSNs‐DNA@SeC treatment (Figure 6D). Taken together, these results indicate that MSNs‐DNA@SeC effectively inhibits SLE‐induced kidney damage and enhances the anti‐inflammatory ability of mice through regulation of immune response in MRL/lpr SLE model mouse (Figure 6E).

## 3 Conclusion

In this study, the synthesized MSNs‐DNA demonstrated the ability to recognize, bind, and remove anti‐dsDNA antibodies in plasma from SLE patients, exhibiting a Ka value significantly higher than that observed in plasma with low anti‐dsDNA titers. Additionally, fluorescently labeled MSNs‐DNA enabled the detection of anti‐dsDNA antibody distribution in the kidneys of LN patients. Overall, this combination strategy, which possesses a favorable safety profile, showed high therapeutic efficacy against SLE in MRL/lpr mice. In future work, we aim to design an immunosorbent column containing MSN‐DNA@SeC and develop a device to replicate this process in a mouse model. The proposed device would use the immunosorbent column to adsorb and remove serum antibodies, followed by the retransfusion of the filtered blood back into the animal.

## 4 Experimental Section

4.1

4.1.1

##### Materials

All acidic solvents were sourced from a local chemical reagent factory (Guangzhou, China). The QUANTA Lite dsDNA ELISA kit was procured from Inova Diagnostics (Werfen, Spain). The calf thymus DNA (ctDNA) and coumarin 6 were obtained from Sigma–Aldrich (Burlington, USA). All experiments were conducted using ultrapure water and purified using a Millipore Milli‐Q water purification system (Burlington, USA).

##### Collection of Plasma and Kidney Biopsy Samples from Patients

This study, conducted from 2017 to 2018, involved a cohort of 125 patients diagnosed with SLE who exhibited positive serum anti‐dsDNA antibodies according to the 1997 American College of Rheumatology (ACR) lupus classification criteria.^[^ ^38^ ^]^ Clinical information and plasma specimens were gathered from these individuals, with kidney biopsy samples obtained from those who underwent renal biopsies. Ethical approval for the study was granted by the Ethics Committee of Sun Yat‐Sen University ([2017]2‐176), and informed consent was secured from all participants.

##### Preparation of MSNs‐DNA and MSN‐DNA@SeC

MSNs and MSNs‐COOH nanoparticles were synthesized according to previous procedures.^[^ ^39^ ^]^ To obtain the MSNs‐DNA nanoparticles, 50 mg of the MSNs‐COOH was added into 10 mL water, then 20 mg of NHS and EDC was added into the mixture and stirred for 2 h, finally 2.5 mg of ctDNA was added and stirred for another 24 h. To obtain the SeC@MSNs‐DNA nanoparticles, 50 mg of the MSNs‐COOH was added into 10 mL of the SeC solution (2 mg mL^−1^) and stirred for 24 h at room temperature. Then, 20 mg of NHS and EDC was added into the mixture and stirred for another 2 h. Finally, 2.5 mg of ctDNA was added and stirred for another 24 h. The MSNs‐DNA and SeC@MSNs‐DNA nanoparticles were then centrifuged and vacuum dried. In all experiments, the concentrations of the SeC@MSNs‐DNA nanosystem were determined based on the Se content.

##### Characterization of MSNs‐DNA

After synthesis, the chemical structure of MSNs‐DNA was characterized using a Zetasizer Nano ZS particle analyzer (Malvern, USA), FT‐IR, and Raman spectroscopy. TEM was further employed to examine the morphology. The concentrations of Se and silicon (Si) in the nanoparticles were quantified through inductively coupled plasma mass spectrometry.

##### Isothermal Titration Calorimetry (ITC)

The measurement of ITC was conducted at room temperature by the Microcal Nano ITC calorimeter (Northampton, USA). The titration of patient serum (anti‐dsDNA‐antibodies) to MSNs‐DNA followed previously established protocols. Data analysis was conducted utilizing the one‐set binding model within Origin software, integrated with the ITC system, to ascertain the affinity constant (Ka) and derive additional thermodynamic parameters. The reported thermodynamic values presented are the averages derived from duplicate experiments.

##### Adsorption of Anti‐dsDNA Antibodies by MSNs‐DNA

A total of 300 μL of plasma and 6 mg of MSNs‐DNA were successively introduced into an Eppendorf tube and agitated for 2 h at room temperature. Subsequently, the mixture underwent centrifugation at 12 000 rpm for 10 min to segregate the anti‐dsDNA antibodies bound to MSNs‐DNA within the supernatant. Quantification of the anti‐dsDNA antibodies was carried out using a dsDNA kit, measuring the values of absorbance at 450 and 620 nm wavelengths on a Bio‐Tek Microplate reader (Winooski, USA). Comprehensive nanoparticle characterization was further conducted using TEM, a Zetasizer Nano ZS particle analyzer, FT‐IR, and Raman spectroscopy. The clearance ratio of anti‐dsDNA antibodies was calculated as follows

(1) $$\text{Titer }\left(\text{Unit}\right)=\frac{\left(A_{\text{OD450}}-A_{\text{OD620}}\right)}{\left(B_{\text{OD450}}-B_{\text{OD620}}\right)}\times 375$$

(2) $$\text{Clearance }\text{ratio }\left(\%\right)=\frac{\left({\text{Titer}}_{B}-{\text{Titer}}_{A}\right)}{\left({\text{Titer}}_{B}\right)}\times 100\%$$

where *A* is a sample and *B* is dsDNA ELISA calibration in (1).

where A is the sample after absorption and B is the sample before absorption in (2).

To further validate the aforementioned results, the clearance ratio of MSNs‐DNA was compared against two commercially available selective plasma component adsorption columns. A suspension containing 10 mg of MSNs‐DNA (20 mg mL^−1^), along with 25 mg of product 1 (50 mg mL^−1^) and product 2 (50 mg mL^−1^) in 500 μL of plasma, was subjected to incubation for varying durations. Subsequently, the supernatant was isolated by centrifugation at 12 000 rpm for 10 min for further analysis of anti‐dsDNA antibodies.

##### Immunofluorescence on Kidney Biopsy Samples Using MSNs‐DNA

Samples obtained from kidney biopsies of patients were prepared by fixation in 4% paraformaldehyde, followed by staining with H&E and immunofluorescence using coumarin 6 labeled MSNs‐DNA. Subsequently, the samples were examined using a Nikon ni‐u light microscope (Japan) and a Life EVOS FL auto fluorescence microscope (American), respectively. The mean fluorescence intensity of the glomeruli was quantified and calculated using Image J software.

##### Biocompatibility of MSNs‐DNA with Patient Plasma

The hemocompatibility and biocompatibility of MSNs‐DNA were evaluated using established methods, with the latter assessed through the analysis of 33 biochemical indices, including AST.^[^ ^40^ ^]^ For instance, a suspension of 10 mg of MSNs‐DNA in 500 μL of plasma was incubated for 2 h. Subsequently, the supernatant was isolated by centrifugation at 12 000 rpm for 10 min for further biochemical index analysis.

##### The Therapeutic Efficacy of MSNs‐DNA in MRL/lpr SLE Model Mouse

All animal experiments were conducted under the approval of the Animal Experimentation Ethics Committee of Jinan University (13232). We purchased female 8‐week‐old MRL/lpr mice from SLAC Laboratory Animal Company (Shanghai, China). Female c57 healthy mouse groups of the same age were injected with an equal dose of saline only, called “Health Control.” MRL/lpr mice were randomized into 4 groups: female MRL/lpr SLE model mice of the same age injected with an equal dose of saline, called “Blank Control,” and MRL/lpr female SLE model mice treated with SeC, MSNs‐DNA, and MSNs‐DNA@SeC for blood transfusion, respectively. And mice were treated with different drugs three times a week from the age of 16–18 weeks. Urine was collected every week until mice became ill, and urine was collected before injection during treatment. At the end of treatment, mice were anesthetized. Spleens, blood, and kidneys were obtained for subsequent analysis.

##### Statistical Analysis

All experiments were performed in triplicate, and the results were expressed as the mean ± standard deviation (SD). A two‐tailed Student's *t*‐test was employed to assess the variances between the control and experimental groups. Statistical significance was deemed at *P* < 0.05 (*) or *P* < 0.01(**).

## Conflict of Interest

The authors declare no conflict of interest.

## Author Contributions

**Ting Liu**: conceptualization (equal); funding acquisition (supporting); investigation (equal); methodology (equal); writing—original draft (equal). **Zhiming Lin**: investigation (equal); writing—review and editing (supporting). **Xi Zhang**: investigation (equal); methodology (supporting). **Yu Yang**: data curation (equal). **Guanning Huang**: methodology (supporting). **Yanzi Yu**: methodology (supporting). **Bin Xie**: methodology (supporting). **Lizhen He**: conceptualization (equal); funding acquisition (equal); writing—review and editing (equal). **Tianfeng Chen**: conceptualization (equal); funding acquisition (equal); supervision (equal); writing—review and editing (equal).

## Supporting information

Supplementary Material

## Data Availability

Research data are not shared.

## References

[1] M. R. Barber , C. Drenkard , T. Falasinnu , A. Hoi , A. Mak , N. Y. Kow , E. Svenungsson , J. Peterson , A. E. Clarke , R. Ramsey‐Goldman , Nat. Rev. Rheumatol. 2021, 17, 515. 34345022 10.1038/s41584-021-00668-1 PMC8982275

[2] A. Fanouriakis , N. Tziolos , G. Bertsias , D. T. Boumpas , Ann. Rheum. Dis. 2021, 80, 14. 33051219 10.1136/annrheumdis-2020-218272

[3] S. Caielli , Z. Wan , V. Pascual , Ann. Rev. Immunol. 2023, 41, 533. 36854182 10.1146/annurev-immunol-101921-042422

[4] A. Fanouriakis , M. Kostopoulou , J. Andersen , M. Aringer , L. Arnaud , S.‐C. Bae , J. Boletis , I. N. Bruce , R. Cervera , A. Doria , Ann. Rheum. Dis. 2024, 83, 15. 37827694 10.1136/ard-2023-224762

[5] G. C. Tsokos , Nat. Immunol. 2020, 21, 605. 32367037 10.1038/s41590-020-0677-6 PMC8135909

[6] Y. Tanaka , Int. J. Rheum. Dis. 2020, 23, 465. 32134201 10.1111/1756-185X.13817 PMC7187183

[7] M. Infantino , E. Nagy , N. Bizzaro , K. Fischer , X. Bossuyt , J. Damoiseaux , Autoimmunity 2022, 5, 100139. 10.1016/j.jtauto.2021.100139 PMC8741517 35028552

[8] M. Bruschi , A. Angeletti , M. Prunotto , P. L. Meroni , G. M. Ghiggeri , G. Moroni , R. A. Sinico , F. Franceschini , M. Fredi , A. Vaglio , Autoimmun. Rev. 2024, 23, 103535. 38552995 10.1016/j.autrev.2024.103535

[9] F. Basta , F. Fasola , K. Triantafyllias , A. Schwarting , Rheumatol. Ther. 2020, 7, 433. 32488652 10.1007/s40744-020-00212-9 PMC7410873

[10] C. Mohan , T. Zhang , C. Putterman , Nat. Rev. Nephrol. 2023, 19, 491. 37225921 10.1038/s41581-023-00722-z

[11] R. Furie , B. H. Rovin , F. Houssiau , A. Malvar , Y. O. Teng , G. Contreras , Z. Amoura , X. Yu , C.‐C. Mok , M. B. Santiago , New England J. Med. 2020, 383, 1117. 32937045 10.1056/NEJMoa2001180

[12] S. V. Parikh , S. Almaani , S. Brodsky , B. H. Rovin , Am. J. Kidney Dis. 2020, 76, 265. 32220510 10.1053/j.ajkd.2019.10.017

[13] J. E. Rojas‐Rivera , C. García‐Carro , A. I. Ávila , M. Espino , M. Espinosa , G. Fernández‐Juárez , X. Fulladosa , M. Goicoechea , M. Macía , E. Morales , Clin. Kidney J. 2023, 16, 1384. 37664575 10.1093/ckj/sfad055 PMC10468759

[14] M. Gasparotto , M. Gatto , V. Binda , A. Doria , G. Moroni , Rheumatology 2020, 59, v39. 33280015 10.1093/rheumatology/keaa381 PMC7751166

[15] C. C. Mok , Y. O. Teng , R. Saxena , Y. Tanaka , Nat. Rev. Rheumatol. 2023, 19, 227. 36864291 10.1038/s41584-023-00925-5

[16] K. Connelly , L. E. Eades , R. Koelmeyer , D. Ayton , V. Golder , R. Kandane‐Rathnayake , K. Gregory , H. Brunner , L. Burke , L. Arnaud , Nat. Rev. Rheumatol. 2023, 19, 592. 37433880 10.1038/s41584-023-00993-7

[17] D. S. Pisetsky , P. E. Lipsky , Nat. Rev. Rheumatol. 2020, 16, 565. 32884126 10.1038/s41584-020-0480-7 PMC8456518

[18] M. Piga , L. Arnaud , J. Clin. Med. 2021, 10, 243. 33440874 10.3390/jcm10020243 PMC7827672

[19] L. Sun , H. Liu , Y. Ye , Y. Lei , R. Islam , S. Tan , R. Tong , Y.‐B. Miao , L. Cai , Signal Transduction Targeted Ther. 2023, 8, 418. 10.1038/s41392-023-01642-x PMC10622502 37919282

[20] A. Joorabloo , T. Liu , Exploration 2024, 4, 20230066. 38939866 10.1002/EXP.20230066 PMC11189585

[21] L. Li , Y. Shen , Z. Tang , Y. Yang , Z. Fu , D. Ni , X. Cai , Exploration 2023, 3, 20220148. 38264689 10.1002/EXP.20220148 PMC10742205

[22] C. Huang , H. Wang , X. Yang , Q. Yu , H. Wang , L. Zhang , Y. Zhao , D. Zhu , Adv. Funct. Mater. 2024, 34, 2401489.

[23] P. Bigini , M. Gobbi , M. Bonati , A. Clavenna , M. Zucchetti , S. Garattini , G. Pasut , Nat. Nanotechnol. 2021, 16, 1169. 34732846 10.1038/s41565-021-01001-3

[24] L.‐X. Chai , X.‐X. Fan , Y.‐H. Zuo , B. Zhang , G.‐H. Nie , N. Xie , Z.‐J. Xie , H. Zhang , Coord. Chem. Rev. 2021, 432, 213697.

[25] G. Genchi , G. Lauria , A. Catalano , M. S. Sinicropi , A. Carocci , Int. J. Mol. Sci. 2023, 24, 2633. 36768955 10.3390/ijms24032633 PMC9917223

[26] W. Ding , S. Wang , J. Gu , L. Yu , Chin. Chem. Lett. 2023, 34, 108043.

[27] T. Filippini , S. Fairweather‐Tait , M. Vinceti , Am. J. Clin. Nutr. 2023, 117, 93. 36789948 10.1016/j.ajcnut.2022.11.007

[28] H. F. Bradford , T. C. McDonnell , A. Stewart , A. Skelton , J. Ng , Z. Baig , F. Fraternali , D. Dunn‐Walters , D. A. Isenberg , A. R. Khan , Nat. Immunol. 2024, 25, 873. 38553615 10.1038/s41590-024-01798-w PMC11065695

[29] R. Shah , B. Ibis , M. Kashyap , V. A. Boussiotis , Metabolism 2023, 151, 155747. 38042522 10.1016/j.metabol.2023.155747 PMC10872310

[30] L. Lan , Z. Feng , X. Liu , B. Zhang , J. Cell. Mol. Med. 2024, 28, e18390. 38801402 10.1111/jcmm.18390 PMC11129730

[31] T. Wei , T. Pan , X. Peng , M. Zhang , R. Guo , Y. Guo , X. Mei , Y. Zhang , J. Qi , F. Dong , Nat. Nanotechnol. 2024, 19, 1178. 38740936 10.1038/s41565-024-01660-y

[32] Y. Du , Z. Zhang , Y. Yang , T. Liu , T. Chen , X. Li , Nanophotonics 2022, 11, 5101. 39634308 10.1515/nanoph-2022-0289 PMC11501141

[33] B. Xianyu , S. Pan , S. Gao , H. Xu , T. Li , Small 2024, 20, 2306225. 10.1002/smll.202306225 38072799

[34] S. Liu , N. Li , H. Lai , L. Xu , Y. Zeng , X. Chen , H. Huang , T. Chen , J. Liu , J. Wang , Adv. Funct. Mater. 2024, 34, 2401264.

[35] E. Carlsson , A. Quist , J. C. Davies , A. Midgley , E. M. Smith , I. N. Bruce , M. W. Beresford , C. M. Hedrich , Clin. Immunol. 2022, 236, 108948. 35123058 10.1016/j.clim.2022.108948

[36] M. Aringer , J. Autoimmune. 2020, 110, 102374. 10.1016/j.jaut.2019.102374 31812331

[37] Q. Zhu , Y. Li , L. Zhang , M. Wang , Z. Chen , J. Shi , J. Li , B. Li , Z. Li , Y. Wang , Clin. Rheumatol. 2021, 40, 151. 32542581 10.1007/s10067-020-05220-2

[38] M. C. Hochberg , Arthritis Rheum. 1997, 40, 1725. 10.1002/art.1780400928 9324032

[39] Y. You , L. He , B. Ma , T. Chen , Adv. Funct. Mater. 2017, 27, 1703313.

[40] S. Pan , G. Huang , Z. Sun , X. Chen , X. Xiang , W. Jiang , Y. Xu , T. Chen , X. Zhu , Adv. Funct. Mater. 2023, 33, 2213364.

