# Supplementary material for: Targeting Nanotherapeutics for Highly Efficient Diagnosis and Treatment of Systemic Lupus Erythematosus through Regulation of Immune Response
Source: Small Sci. 2025 Jan 23;5(5):2400521. doi: 10.1002/smsc.202400521 (PMC12087781; doi:10.1002/smsc.202400521)
Supplement: Supplementary file 1 — Supplementary Material [file SMSC-5-2400521-s001.pdf]

Supporting information

For

Targeting Nanotherapeutics for Highly Efficient Diagnosis and Treatment of Systemic Lupus Erythematosus through Regulation of Immune Response

Ting Liu[1], Zhiming Lin[2], Xi Zhang[2], Yu Yang[1], Guanning Huang[1], Yanzi Yu[1], Bin Xie[1], Lizhen He[1]*, Tianfeng Chen[1]*

[1] The Department of Chemistry, Department of Neurology and Stroke Center of The First Affliated Hospital, State Key Laboratory of Bioactive Molecules and Druggablily Assessment, MOE Key Laboratory of Viral Pathogenesis & lnfection Prevention and Control, Jinan University, Guangzhou 510632, China

[2] Division of Rheumatology, Third Affiliated Hospital of Sun Yat-Sen University, Guangzhou 510630, China

No conflict of interest was reported by the authors of this article.

* Corresponding Author: Dr. Tianfeng Chen, Email: tchentf@jnu.edu.cn; Dr. Lizhen He, Email: hlz6371@jnu.edu.cn.

Results

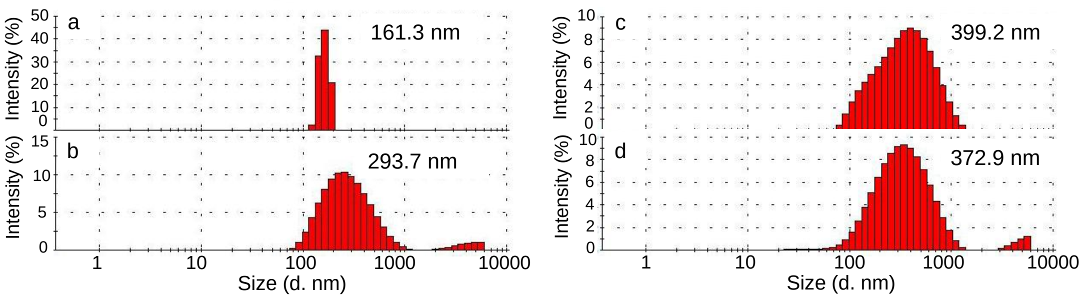

Figure S1. Size distribution of MSNs-DNA after adsorbed anti-dsDNA antibodies in plasma of SLE patients. (a: before adsorbed the anti-dsDNA antibodies, b, c and d: after adsorbed the anti-dsDNA antibodies in plasma of different SLE patients.)

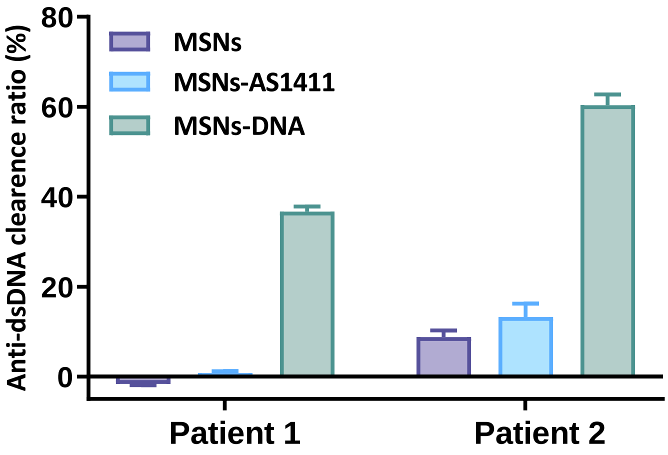

Figure S2. The anti-dsDNA antibody clearance ratio of MSNs, MSNs-AS1411 and MSNs-DNA. Each value represents means ± SD (n=3).

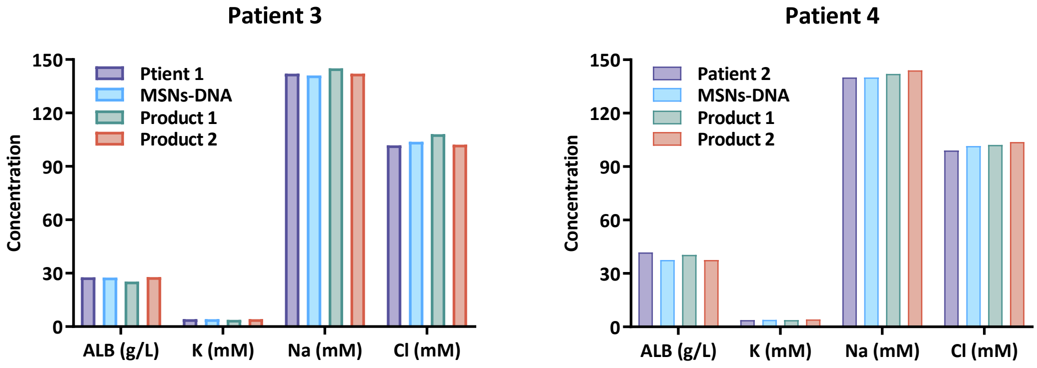

Figure S3. Safety evaluation of MSNs-DNA. The ALB, Na, K and Cl in plasma of **(A)** patient 3 and **(B)** patient 4 before and after adsorption.

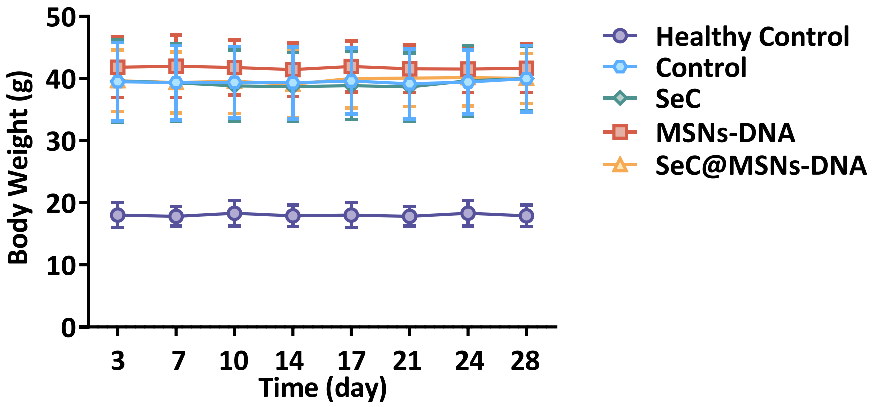

Figure S4. The body weight of mice with different treatments.

Table S1. Titer before and after absorption and clearance ratio of anti-dsDNA antibodies in 125 SLE patients.

Patients	Titer (before)	Titer (after)	clearance ratio (%)
1	10.24668	9.748576	4.861111
2	33.05554	8.053890	75.63527
3	56.2667	36.20325	35.65776
4	57.79258	26.07184	54.88721
5	70.06901	17.72595	74.70214
6	91.70184	26.84334	70.72758
7	240.2557	109.0491	54.61121
8	257.0446	12.09677	95.29390
9	261.1587	26.46371	89.86680
10	272.0948	26.65850	90.20249
11	272.4508	64.74507	76.23604
12	285.8415	50.47862	82.34034
13	290.2665	60.39976	79.19161
14	290.4357	76.43371	73.68308
15	293.6941	176.0178	40.06762
16	307.5658	30.81554	89.98082
17	311.8043	22.51224	92.78000
18	313.8654	87.14001	72.23650
19	323.4235	192.6204	40.44329
20	329.1336	99.77035	69.68696
21	336.3268	51.70915	84.62532
22	340.0261	93.19141	72.59286
23	342.6639	75.09930	78.08368
24	343.1656	287.6224	16.18553
25	347.4739	120.7485	65.24962
26	353.2735	87.34067	75.27675
27	362.1524	31.09483	91.41388
28	367.613	40.41135	89.00709
29	370.6547	52.57821	85.81477
30	379.3453	67.35225	82.24513
31	380.2144	87.34067	77.02857
32	390.6431	69.09038	82.31368
33	402.3754	104.7219	73.97408
34	402.8829	29.77515	92.60947
35	411.898	119.6933	70.94100
36	412.4876	72.55461	82.41047
37	416.4287	163.3254	60.77949
38	418.2714	47.44545	88.65677
39	428.4561	38.76335	90.95278
40	434.7071	161.4635	62.85693

41	461.5038	77.56166	83.19370
42	463.9898	184.0805	60.32658
43	472.1537	131.6413	72.11895
44	474.0566	231.3493	51.19795
45	476.2355	76.66406	83.90206
46	476.2457	95.59675	79.92700
47	477.9978	227.8426	52.33396
48	482.8631	52.68073	89.08992
49	485.3178	107.5426	77.84077
50	487.8041	121.4001	75.11292
51	493.7667	47.75155	90.32912
52	500.931	159.3532	68.18857
53	501.0062	31.03195	93.80607
54	534.0884	38.26246	92.83592
55	541.0253	245.5623	54.61167
56	545.336	397.1610	27.17131
57	547.0742	101.2456	81.49324
58	550.9868	238.9523	56.63193
59	551.0674	183.7998	66.64658
60	556.2145	281.3280	49.42094
61	562.7173	152.9548	72.81853
62	565.3244	116.8887	79.32359
63	567.9263	143.6164	74.71213
64	570.8482	71.29341	87.51096
65	572.2769	116.8887	79.57479
66	586.6165	131.2282	77.62962
67	590.4175	99.92884	83.07488
68	591.5085	81.47533	86.22584
69	594.8725	83.42989	85.97516
70	600.0869	95.16222	84.14192
71	600.6641	151.0436	74.85389
72	603.5632	239.8609	60.25917
73	604.9644	77.38757	87.20791
74	612.649	202.1474	67.00435
75	614.0454	189.2378	69.18178
76	622.2173	37.17720	94.02504
77	625.0621	149.9813	76.00536
78	626.9861	228.0598	63.62601
79	633.7761	519.2125	18.07634
80	636.153	275.4924	56.69398
81	645.7126	370.2201	42.66487
82	656.6414	193.7144	70.49920
83	676.9213	131.2618	80.60899
84	678.8457	121.7442	82.06599

85	686.9414	305.7969	55.48427
86	690.3662	70.79252	89.74565
87	692.9791	208.5388	69.90690
88	701.0911	118.4060	83.11117
89	705.5321	100.6789	85.73006
90	711.7647	182.8747	74.30685
91	712.2952	164.9702	76.83962
92	714.1585	113.0342	84.17238
93	716.5411	210.3128	70.64887
94	718.8567	278.0123	61.32576
95	724.9833	576.0240	20.54657
96	738.3937	452.6750	38.69462
97	741.4374	300.3083	59.49646
98	751.8027	230.7784	69.30332
99	756.5479	111.1283	85.31112
100	760.4909	133.0142	82.50942
101	761.5275	352.3244	53.73450
102	767.9791	261.7172	65.92130
103	770.4222	264.9430	65.61066
104	772.1611	314.6002	59.25717
105	776.0185	74.71616	90.37185
106	785.1458	94.50133	87.96384
107	787.0842	420.9285	46.52052
108	788.986	83.48174	89.41910
109	798.2329	271.5816	65.97713
110	801.3046	118.9509	85.15533
111	832.6403	113.7971	86.33297
112	841.4125	136.1308	83.82114
113	856.5227	248.2468	71.01689
114	884.2943	185.8581	78.98231
115	893.2268	213.1567	76.13632
116	901.2848	401.4709	55.45570
117	902.5203	228.5631	74.67501
118	908.3092	70.26380	92.26433
119	919.0227	397.1226	56.78859
120	919.0324	102.9837	88.79432
121	930.7936	236.3368	74.60910
122	949.5492	257.3185	72.90097
123	967.0304	421.0863	56.45572
124	986.8192	26.50637	97.31395
125	987.0603	335.9305	65.96656

Table S2. The change of anti-dsDNA antibodies titer (before and after absorption) in 125 SLE patients.

Titer scope	Patients numbers/raito Before absorption	Patients numbers/raito After absorption
<200	6 (4.8%)	89 (71.2%)
200-400	26 (20.8%)	30 (24.0%)
400-600	37 (29.6%)	6 (4.8%)
600-800	40 (32.0%)	0
>800	16 (12.8%)	0

Table S3. Monitoring of urine protein during treatment in MRL/lpr SLE model mouse.

Urine protein (mg/dL)	Time (Day)	1	2	3	4	5	6
Healthy Control	0	30	30	30	30	30	30
	7	30	30	40	30	30	30
	14	40	30	10	30	40	40
	21	30	20	10	30	30	30
	28	40	10	10	30	30	10
Control	0	30	30	30	100	30	30
	7	30	30	40	100	30	30
	14	40	30	100	200	40	40
	21	30	20	100	200	30	30
	28	40	100	100	200	30	100
SeC	0	100	30	30	30	30	30
	7	300	30	300	100	30	30
	14	300	30	300	100	30	30
	21	300	30	200	100	40	30
	28	300	30	200	100	30	100
MSNs-DNA	0	30	100	30	30	30	30
	7	30	100	100	100	100	30
	14	40	100	100	100	100	30
	21	40	100	100	40	100	30
	28	30	200	200	100	40	30
SeC@MSNs-DNA	0	200	30	30	30	30	30
	7	300	30	30	100	30	30
	14	100	30	10	30	10	10
	21	100	10	10	30	10	10
	28	100	30	30	100	30	10
